# Establishment of radiation therapy services in North West Tasmania: a community need or election strategy?

**DOI:** 10.1186/s12913-019-4085-2

**Published:** 2019-04-24

**Authors:** Sancia West, Elizabeth Shannon, Elaine Crisp, Tony Barnett

**Affiliations:** Centre for Rural Health, School of Health Sciences, University of Tasmania, Locked Bag 1372, Launceston, Tasmania 7250 Australia

**Keywords:** Radiation therapy, Policy change, Advocacy coalition framework, Rural health services, Document analysis

## Abstract

**Background:**

This case study examines the impetus for policy change that resulted in the establishment of a radiation therapy service in rural North West Tasmania, Australia. Provision of local radiation therapy services improves accessibility for those in rural and regional areas. However, providing these services and maintaining them is not achievable for all areas. The drivers to establish services in more regional locations are not always well understood.

This article presents a case study of how a radiation therapy service was established in North West Tasmania. It applies a health policy analysis model (the Advocacy Coalition Framework) to examine the impetus for policy change and draws conclusion about how the framework can be applied to the development of health services in rural areas.

Understanding the impetus for policy change allows health service planners to apply this knowledge to influence the health agenda. Knowing the way in which policy change can be driven creates an opportunity to become more strategically involved in policymaking.

**Method:**

Documents related to the case study were analysed for expressed beliefs, using the Advocacy Coalition Framework, to determine any identifiable coalition of actors that held consistent, shared beliefs and were engaged in non-trivial action to the establish radiation therapy services in North West Tasmania.

**Results:**

Document analysis confirmed the presence of a Health Policy Coalition that was concerned about sustainability and safety in establishing the service. No additional coalition was identified. Instead, the possible role of the media and the marginal nature of the local Federal electorate were likely to have impacted the subsequent policy change.

**Conclusions:**

The study found evidence that policy change was achieved primarily as a result of a political strategy designed to win support during a Federal election. This has important implications for health policy in rural areas, especially for those population centres located in marginal seats. During an election cycle the decision to establish new health services may not be wholly influenced by an identified coalition or issue such as sustainability, community needs or rationality.

## Background

North West (NW) Tasmania, Australia, has a population of approximately 91,000 [1] and is ranked as one of the most socioeconomically disadvantaged areas in Australia [2]. Prior to 2016 there was no locally based radiation therapy service available to cancer patients in the NW, with most patients being referred to Launceston, approximately 100-160 km from the regional centres of Devonport and Burnie. Anecdotal reports suggested some patients were unwilling or unable to travel for treatment and subsequently made the decision to receive the level of treatment available in their local area, even if this did not offer them the best chance of survival [3–5]. The opening of the NW Regional Cancer Centre (the Centre) in 2016 brought locally-based radiation therapy services to the region for the first time and was achieved as a direct result of funding commitments made during the 2010 Federal Election.

The NW has a long history of local provision of health services. Hospitals in the region have historically operated autonomously and benefitted from a high degree of public support [6]. Attempts to change the service mix, or downgrade existing services have been met with strong reactions from the local community [7, 8]. This concern for retaining local services has long been a political tool [9, 10], exacerbated by the fact that the local Federal electorate of Braddon is highly marginal.

As part of the 2007 Federal Election campaign, the Labor Opposition pledged $7.7 million to fund a linear accelerator (LINAC) for radiation therapy in Northern Tasmania, with the North West a possible site [11]. However, the decision was ultimately made to install a third LINAC in Launceston [12]. The Braddon Member called this “a broken promise”, indicating the level of sentiment felt within the NW community after the funding went elsewhere [11]. This incident may have been a catalyst for increased public focus on this issue in subsequent years [13].

Media discussion on the need of a North West based radiation therapy service increased sharply in the twelve months up to the 2010 Federal Election. The Liberal Opposition responded to this by committing $7 million for a LINAC in Burnie [14]. The Federal Labor Government then committed $16.5 million for a LINAC and infrastructure [11], which ultimately led to the building of the Centre upon Labor’s re-election.

Understanding the imperatives that drive policy change in health services for rural and remote areas is integral to achieving better health outcomes for these communities. This study applied the Advocacy Coalition Framework (ACF), a method for longitudinal policy analysis [15], to examine reasons behind the change in policy that resulted in the establishment of the Centre in North West Tasmania. It was hypothesised that possible reasons included an evidenced medical need, a political strategy designed to win votes, or sustained and coordinated community pressure.

## Methods

Document analysis was used to identify the actors involved in the policy debate over cancer services in North West Tasmania as articulated by their beliefs, interests and policy positions. Documents relating to cancer services in Tasmania were limited to the year 2000 onwards. With the funding commitment for the Centre – a cornerstone for accessibility of cancer services in North West Tasmania – being announced in 2010, this timeframe gives a full decade to determine the consistency and stability of coalitions in the subsystem, as per the ACF [16]. Literature was sourced and screened for suitability, using set inclusion and exclusion criteria (see Table 1). This ensured information was gathered and assessed in a consistent manner. The adoption of the ACF prior to data collection provided rigour by allowing for a consistent meaning and significance to be placed on information [17].

**Table 1:** Inclusion and Exclusion Criteria

	Inclusion Criteria	Exclusion Criteria
Time Period	2000 – onwards	Before 2000
Language	English only	Non-English
Cancer Type	All other cancer types	Non-melanoma and non-recurrent, non-metastasized melanoma skin cancers
Treatment Type	Surgical, medical oncology, radiation therapy	Screening, vaccination, prevention, education, alternative therapies
Location	Within Tasmania or relevant to the provision of treatment to Tasmanians	Cancer services provided in another state for populations other than Tasmanians

**Fig. 1:**
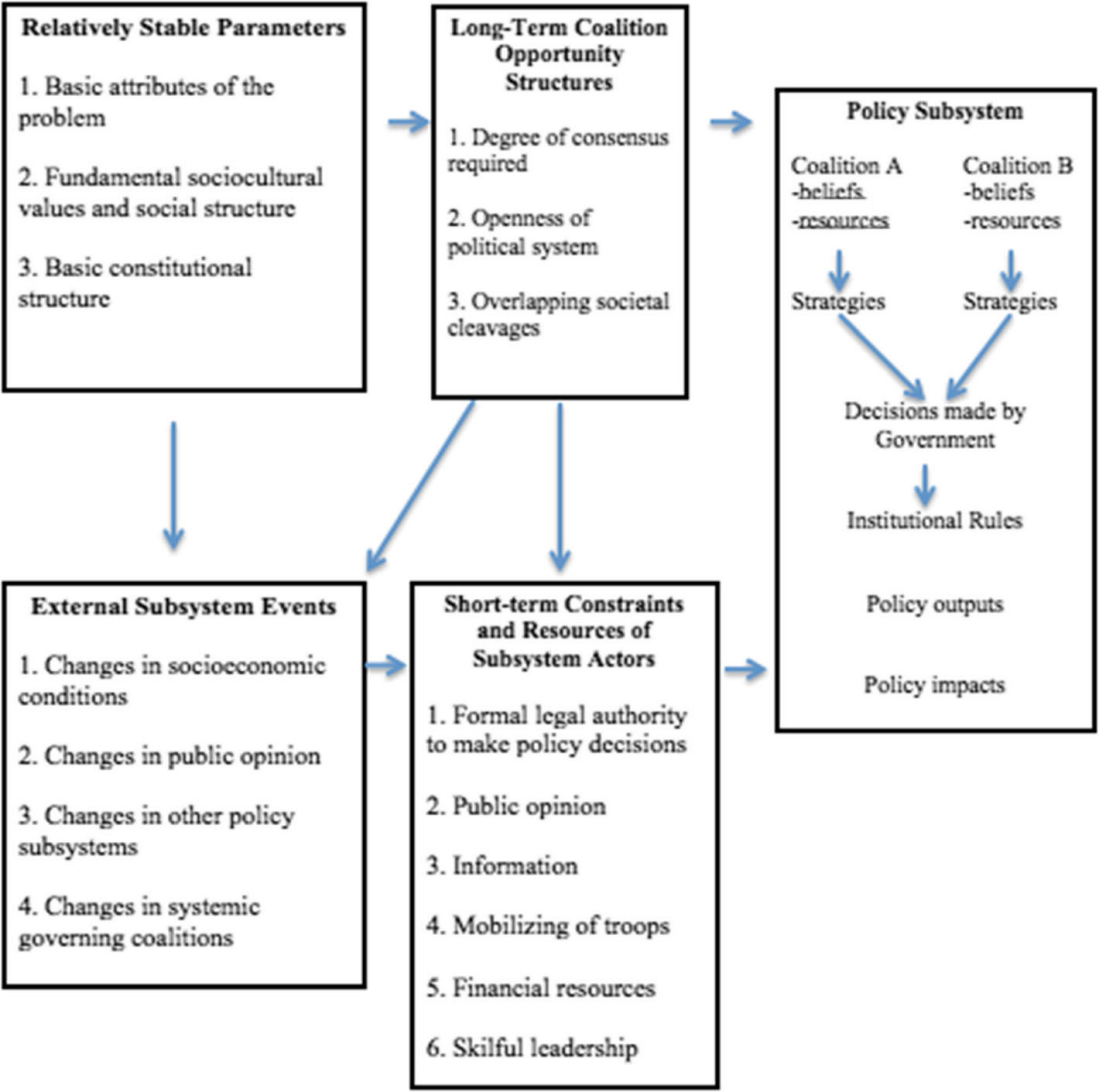
Advocacy Coalition Framework Flowchart

Under the ACF, actors come together in a policy subsystem that is impacted and defined by four factors: the relatively stable parameters that define the nature of the problem; external subsystem events, being impacts outside the particular policy area that may impact upon it; long-term opportunity structure; and the short-term constraints and resources of the actors (see Fig. 1). Actors group themselves according to beliefs into coalitions. These coalitions join the policy debate against other coalitions, mediated by policy brokers, which interact over long periods of time, usually a decade or more, and compete for influence over policy.

Policy change is identified as being triggered in four ways. These include: changes or shocks to the external environment; policy-oriented learning; internal shocks due to the failure of current practices; and alternate dispute resolution, brought about by stalemates, member commitment, and leadership [18].

**Fig. 2:**
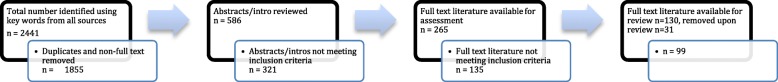
The process for sourcing and retrieving literature

A database search, (EBSCO and ProQuest) using the search terms ‘*cancer + Tasmania + policy + regional’* was used in conjunction with search engines, libraries and citation searches (see Fig. 2). The review focused on full text literature that was most relevant to the provision of cancer treatment services in Tasmania or for Tasmanians or relevant to the health system in Tasmania. The literature reviewed included journal articles and edited books on the ACF, government and non-government reports and documents, Hansard, media articles, policy documents, and media releases and statements made by political candidates, representative bodies and other stakeholders. Abstracts and Executive Summaries were read to confirm how consistently they met the inclusion criteria, creating a final set of documents for analysis.

The total number of documents retrieved meeting the criteria was 130 and analysis of each was coded using the qualitative analysis software NViVO. From this, a further 31 were excluded as no beliefs were expressed. This brought the total number of included documents to 99. All coding was created and crosschecked by all members of the Research Team. This ensured that no systematic error was included in the creating of codes (nodes) in NVivo by a single coder.

Document analysis focused on extracting words, phrases and passages of text that reflected the views, actions and underlying beliefs of these major actors. These beliefs were then ascribed a value – Deep Core, Policy Core, and Secondary Aspects – based on the classifications outlined in Sabatier [19] and Jenkins-Smith and Sabatier [20]. Key beliefs were determined through stated objectives, language used, repetition and therefore salience of certain words, phrases or concepts, and the importance placed on certain outcomes.

Prevalence of these beliefs was mapped over four time periods: from 2000 to the 2007 Federal Election; 2007 to the 2010 Federal Election; 2010 to the opening of the Centre in late 2015; and from 2015 onwards. Separation into discrete and distinct periods allowed the major changes in the policy subsystem to be used as markers to identify whether beliefs and actions of major actors stayed consistent over time and therefore suggested the presence of a coalition.

## Results

Document analysis focused on the actions and statements of major groups in the policy debate in order to gauge their beliefs and to assess the evidence for the existence of a hypothesised coalition stemming from these actor groups. Five major actor groups were identified: Federal Health Policy Actors (FHPA); State Health Policy Actors (SHPA); Health Professionals (HP); Community and Community Advocates (CCA); and the Media. The frequency of their profile of belief (the number of documents) was mapped across the four periods (see Table 2).

**Table 2:** Number of documents by actor group and period

	Pre-2007	2007–2010	2010–2015	2015 onwards
Federal	3	9	7	1
State	3	16	25	1
Health Prof	9	5	12	0
Community	6	9	9	3
Media	1	6	4	2

(*n* = 99 documents, 131 instances of group beliefs)

The analysis showed that there was no single belief that was consistently demonstrated across all four periods by any group (see Table 3). However, there were several instances of a belief being expressed by a group of actors across three consecutive time periods:

**Table 3:** Key beliefs by actors and time period

Belief	Level	Pre 2007	2007–10	2010–15	2015/16
Coordination of care	PC	F, H, C	S	S, H	
Support	SA	F, C	S	H, M	
Travel, Transport & Accommodation (as a solution)	PC	H	F, S, H, C	S, H	
Travel, Transport & Accommodation (as a burden)	PC	M	C	C	C
Safety and sustainability	DC	S, H	F, S, H	F. S	
Centralisation versus maintenance of services	SA	H, C			M
Multidisciplinary Care (MDC)	SA	H	S		
Equity & access	DC	F, S, H	C	C	S, C, M
Politically-driven change	PC			H, C	M
Staff skills	SA	F		S, M	
Duplication of services	SA	S		H	
Recruitment & retention of staff	SA	S, H	S, H	S, H, M	
Health budgets	SA	S	F	H	F
Community expectations	PC		F, C, M	F, S, C, M	C
Urgency of the issue	PC		S	C	

F = Federal Health Policy Actors: all stakeholders working or operating at a Federal level in the debate, design or implementation of cancer services affecting Tasmania

S = State Health Policy Actors: all stakeholders working or operating at a State level in the debate, design or implementation of cancer services affecting Tasmania

H = Health Professionals: those working in a health profession or as part of an organisation that represented a health profession. This includes medical professionals, nurses and allied health professionals

C = Community and Community Advocates: members of the public or organisations or groups whose role was to advocate on behalf of the community or cancer patients

M = Media: those employed in the media industry who provided comment relating to cancer services in Tasmania as part of their occupation, or media outlets themselves where no reporter or author was identified

*DC* Deep Core, *PC* Policy Core, *SA* Secondary Aspects

SHPA

- Safety and sustainability – Periods 1–3
- Recruitment and retention – Periods 1–3

HP

- Travel, transport and accommodation (as a solution) – Periods 1–3
- Safety and sustainability – Periods 1–3

CCA

- Travel, transport and accommodation (as a burden) – Periods 2–4
- Community expectations – Periods 2–4
- Equity and access – Periods 2–4

Table 2 demonstrates that there was no belief consistently demonstrated across three or more periods by FHPA. When the issue of radiation therapy began to gain traction in 2007, after the first funding commitment for a linear accelerator for the North was made, discussion focused on safety and sustainability as well as community expectations. These were the only two beliefs to be discussed in more than one period. These dissipated after the opening of the Centre in late 2015.

SHPA show a more consistent and developed set of beliefs being discussed, perhaps attributable to the local nature of the issue. Safety and sustainability and recruitment and retention were demonstrated across three consecutive periods. Travel became another focal point as the debate over radiation therapy services and the best design for delivering this progressed. These beliefs centre on a practical consideration of administration and resources, in keeping with the nature of this actor group, and were supported by consistent action in the form of health reform initiatives and public statements.

The discussion by HP demonstrated two key beliefs: travel; and safety and sustainability. Travel was discussed from the perspective of this being a solution to the issue of accessibility for regional and remote patients. The group also made repeated public statements regarding recruitment and retention during the 2010–2015 period after the Centre became a funding reality. This shows a similar focus to that of the SHPA, being resource and administration issues.

The CCA groups had three core beliefs, demonstrated across three consecutive periods. These focused on travel; equity and access; and community expectations. These represented a focus on individuals and their experience of accessing cancer services, rather than resources or administrative matters. Travel was discussed from the perspective of this being a burden for patients, rather than as a solution to accessibility.

The Media group had the least consistently demonstrated beliefs, with only one belief – community expectations – being discussed in two consecutive periods. However, during the period of 2007–2015 the media were at time prolific in generating articles relating to the issue of cancer services in the North West. Their focus on community expectations is in line with the perceived role of the media as representing the views of the readership.

Overall, this provides preliminary evidence of a possible Health Policy Coalition (HPC) comprised of SHPA and HP. This coalition was largely opposed to a local radiation therapy service. The belief of safety and sustainability was shared consistently across a period of more than a decade, and non-trivial action was evident, including consistent public statements and efforts to promote sustainable health reform. However, there was no clear evidence from the document analysis of any competing coalition advocating consistently for a regional radiation therapy service, coming together in non-trivial action based on policy beliefs. There were numerous statements made in media articles expressing that the community lobbied continuously for an extended cancer service but no evidence of this could be found in the documents retrieved for analysis.

## Discussion

The key research question is what was the impetus for policy change that resulted in the establishment of the Centre in North West Tasmania? Change was brought about by the force of epidemiological evidence that resulted in policy learning of the need for an extended regional service, a political strategy employed to win votes in a highly marginal Federal electorate, or as a result of sustained pressure from a coordinated community coalition for such a service. The evidence suggests that there was a disjoint between some community opinions that travel was onerous and counterproductive to treatment and some medical opinions that the North West was not large enough to provide a safe and sustainable service.

### Epidemiological evidence

There was little evidence to suggest that policy change was brought about in response to the weight of epidemiological evidence demonstrating the need for a radiation therapy service in the North West. The Australian Medical Association (AMA) was consistently opposed to the introduction of the service due to long-standing issues with recruiting and retaining specialist staff in the region [21, 22]. Additionally, mapping of distances travelled to radiation therapy services found that North West Tasmania fared comparably well to other states [23]. One review of cancer service delivery to rural and remote communities specifically excluded Tasmania as it lacked the extent of remoteness found in other states [24]. The Clinical Expert Panel Report [1] did find that a service in the North West was viable in terms of patient numbers but it found no evidence that travel to Launceston for radiation therapy was unsustainable or onerous, beyond the anecdotal. Whilst demand from North West patients would increase a standalone service in the North West was believed to be unsustainable.

### Political strategy

Policy change can result from political strategy. The ACF’s second policy change hypothesis states the “policy core attributes of a government program in a specific jurisdiction will not be significantly revised as long as the subsystem coalition that instated the program remains in power within that jurisdiction— except when the change is mposed by a hierarchically superior jurisdiction” [18]. Tasmania’s heavy reliance on Commonwealth funding, due to having the second lowest capacity to raise revenue in Australia [6], creates an opportunity for tied Commonwealth funding to be used to bolster - or even override - the health policy of the State Government [25]. For example, the Mersey Community Hospital, located in the North West, was a state-owned and run hospital, but became a Federal election issue and was ‘taken over’ by the Federal Government [8, 10]. Radiation therapy services too were the responsibility of the State Government but became a Federal election issue. Federal funding was committed but the State Government was then required to apply for the funding and provide additional funds for the fit-out and ongoing operational costs of the Centre, despite having only installed a third linear accelerator in Launceston. So the same State jurisdiction remained, however policy change, it could be argued, was imposed hierarchically in an external shock to the policy subsystem.

### Community pressure

The third explanation is effective lobbying by a coalition of community leaders. The ACF is premised on the notion of there being two or more coalitions that compete for influence in the policy subsystem [18]. Yet the document analysis shows a poorly defined mix of actors and possible coalitions. Those opposed to local radiation therapy services were clearly identified and described as an HPC. The coalition in support of these services is less clear. There was a petition drafted by a member of the community and presented to State Parliament. This same person facilitated the public forum on the issue and one journalist, a reporter for the local newspaper, wrote at least 18 known articles framing the issue as one of great importance to the North West [26–29]. However, document analysis, including local media articles, does not identify any coalition of community leaders, or any other person who is clearly and consistently associated with the push for radiation therapy in the region, despite this policy change being achieved. This does not lend support to the notion that policy change was achieved in response to a coalition of community leaders.

Although only one coalition is readily identifiable, the ACF can still shed light on the process of policy change in such instances. One of the assumptions of the ACF is that “the set of relevant subsystem actors includes any person regularly attempting to influence subsystem affairs” [18]. This leads to the question of whether change was agitated for by something or someone other than a consumer coalition, possibly a policy entrepreneur.

### Policy entrepreneurs

The concept of the policy entrepreneur [30, 31] has been increasingly integrated into discussion of the ACF. Policy entrepreneurs challenge the status quo by building a groundswell of support and a body evidence for the need for change. The strategic framing and management of the issue by the policy entrepreneur can mobilise the support needed for change and bring together a range of actors who might otherwise have been disengaged from the debate. This may explain why the idea of radiation therapy in North West Tasmania had no clear lobby group and no groundswell of support until it was presented as an election issue through a petition, public forum and increasing news coverage. This also raises the possible role of the media as policy entrepreneurs in framing and promoting the issue of radiation therapy services, and thereby harnessing community support.

## Limitations

This descriptive case study was relevant to one particular health policy decision that was related to one geographic area. The findings are therefore limited and cannot be generalised. This study did not attempt to compare similar health policy issues or coalition memberships across different geographic areas to determine if they resulted in different or similar outcomes. It also did not seek to compare this case study with other international examples to understand the impact of cultural context or differing forms of government on the formation of health policy responses to this issue. The case study remains relevant as an examination of the impact of political messaging on health policy development, which future research can use as a basis for further comparison, hypothesis testing and development of the ACF.

## Conclusion

Document analysis can provide the opportunity to examine the consistency of actors and beliefs over time, which allows the establishment of coalitions to be determined. What this document analysis shows was that the medical profession consistently opposed expanded cancer services in the region on the basis of concerns around sustainability of a specialist workforce and, therefore, patient safety. Political representatives had a less consistent policy position, moving between the Federal Member for Braddon stating the region needed “a lot more investment” [11] before a new service could be sustained, to a full commitment for this service.

The policy position of the community is less certain still, however, with no clearly defined lobby group from which to assess values. To fully understand this issue and fill in gaps in evidence, interviews with key stakeholders and community members are required. These could provide the opportunity to ascertain the level of interest from the community and the role played by individuals.

Longitudinal policy analysis, through document analysis, provides keen insights into the motivation behind the provision of radiation therapy services in North West Tasmania. This case study therefore has important implications for health policy in rural areas.

## Abbreviations

ACF: Advocacy Coalition Framework
AMA: Australian Medical Association
CCA: Community and Community Advocates
FHPA: Federal Health Policy Actors
HP: Health Professionals
HPC: Health Policy Coalition
LINAC: Linear Accelerator
NW: North West
SHPA: State Health Policy Actors
